# ﻿Systematics of *Lepidocyrtinusboneti* Denis, 1948 (Collembola, Seirinae) reveals a new position for the species within Seirinae

**DOI:** 10.3897/zookeys.1152.99161

**Published:** 2023-03-07

**Authors:** Nerivania Nunes Godeiro, Yun Bu, Areeruk Nilsai, Louis Deharveng, Nikolas Gioia Cipola

**Affiliations:** 1 Shanghai Natural History Museum, Shanghai Science & Technology Museum, Shanghai 200041, China Shanghai Natural History Museum, Shanghai Science & Technology Museum Shanghai China; 2 Division of Biological Science, Faculty of Science, Prince of Songkla University, Hat Yai, Songkhla, 90110, Thailand Prince of Songkla University Songkhla Thailand; 3 Institut de Systématique, Evolution, Biodiversité (ISYEB) – UMR 7205 CNRS, MNHN, UPMC, EPHE, Museum national d’Histoire naturelle, Sorbonne Universités, 45 rue Buffon, CP50, F-75005 Paris, France Sorbonne Universités Paris France; 4 Laboratório de Sistemática e Ecologia de Invertebrados do Solo, Instituto Nacional de Pesquisas da Amazônia—INPA, CPEN, Manaus, AM, Brazil Laboratório de Sistemática e Ecologia de Invertebrados do Solo, Instituto Nacional de Pesquisas da Amazônia—INPA Manaus Brazil

**Keywords:** Asian springtails, Entomobryidae, mitogenome, paraphyletic genus, phylogeny, review, taxonomy

## Abstract

*Seiraboneti* Denis, 1948, **comb. nov.** is examined and redescribed based on syntypes and by a newly discovered Chinese population. Lectotype and paralectotypes were designated, and the type locality of the species has been fixed to Câuda, near Nhatrang, Vietnam. The species was first described in the genus *Lepidocyrtinus*, but based on morphological and molecular evidence it is here transferred to *Seira*. For the phylogenetic placement of *Seiraboneti***comb. nov.**, its mitogenome was included in a dataset comprising 19 species of Seirinae. Maximum Likelihood and Bayesian inferences clustered the species next to *Seirasanloemensis* Godeiro & Cipola, 2020 from Cambodia, forming a distinct *Seira* clade from the Old World, confirming the hypothesis of the existence of a different basal lineage of Seirinae in Southern Asia.

## ﻿Introduction

Understanding the patterns of species diversity is a major goal for most of the researchers studying evolutionary biology. With this aim, an extensive comparison of phenotypic attributes across taxa and well-corroborated phylogenies are necessary (Simon et al. 2019). Seirinae Yosii, 1961 (sensu Zhang and Deharveng 2015) is an Entomobryidae subfamily with currently ca. 230 species in three genera (Bellinger et al. 1996–2023). The first published molecular phylogeny regarding the Seirinae was based on three genes: two mitochondrial and one nuclear, and only three species of *Seira* Lubbock, 1870 and one of *Lepidocyrtinus* Börner, 1903 were sampled (Zhang et al. 2014). This study supported the Seirinae monophyly and a closer relationship of the subfamily with Lepidocyrtinae. Recently, these results were corroborated by a phylogeny based on morphological and barcode data (Zhang et al. 2019), as well as two studies with complete mitogenomes including 26 terminal taxa of the Entomobryidae (Godeiro et al. 2020a, 2021). Robust phylogenetic studies including the Seirinae are still scarce. Despite including representatives of the three current genera of Seirinae, these studies were focused only on species from the Neotropical region (Godeiro et al. 2020a, 2021), not sufficient to define infrageneric relationships. Only with the inclusion of species from several biogeographic regions we can have a clearer overview of the internal organization of the subfamily. In a study including data from an Asian Seirinae species in a phylogeny based only on mitogenomes from New World species, the Asian species appeared as a basal taxon to the entire New World group of Seirinae (Godeiro et al. 2020b).

The Asian continent has a great diversity of Seirinae species, whose species have been described during the last 80 years, but the morphological data originally described are mostly not sufficient for their comparison with other species (Denis 1948; Yosii 1959, 1961b; Yoshii and Suhardjono 1992; Bellinger et al. 1996–2023). Among the Asian taxa, *Lepidocyrtinusboneti* Denis, 1948 was described from Vietnam and Cambodia based on a few morphological characteristics such as the body color pattern, measurements of the body and appendices, bothriotricha pattern of the second to fourth abdominal segment, and morphology of the eyes and scales (Denis 1948). This species was never taxonomically revised, and it is only possible to know from the original description that it belongs to Seirinae due to the presence of heavily ciliated scales on body, the fourth abdominal segment with three bothriotricha, and the mucro falcate without a basal spine (Yoshii and Suhardjono 1992; Soto-Adames 2008; Cipola et al. 2018a, 2020; Zhang et al. 2019; Godeiro et al. 2020a). A systematic study is necessary to reveal its position among the current Seirinae genera, as well as to provide a revision of its diagnostic characters for future interspecific comparisons.

Here we present a systematic study of *Lepidocyrtinusboneti* Denis, 1948 based on the lectotype and paralectotypes designated from syntypes, the species redescription, and a new record from China. We also transfer the species to *Seira* after a phylogenetic study using 19 other mitogenomes of Seirinae.

## ﻿Materials and methods

### ﻿Taxa sampling, sequencing, and mitogenome assembly

The sequenced specimen belongs to the Chinese population of *S.boneti* comb. nov. and it was collected by NNG in October 2021 using an entomological aspirator. One specimen preserved in absolute alcohol was sent to Shanghai Yaoen Biotechnology Co., Ltd, China, where the DNA was extracted using TIANamp Micro-DNA extraction kit (Tiangen Co., Ltd, China). Libraries were constructed using KAPA Hyper Prep Kit (Roche) following custom procedures. Illumina NovaSeq platform was used to produce paired-end reads with 150 bp length. Approximately 10 Gb of data was delivered. NOVOPLASTY v. 3.8.3 (Dierckxsens et al. 2016) was used to assemble the mitogenome with kmer value = 33. MITOZ v. 2.4-alpha (Meng et al. 2019) was used to annotate and visualize the mitogenome. The final mitogenome sequence was submitted to the NCBI database, and the accession number is listed in Table 1.

**Table 1.** Taxonomical information and GenBank accession numbers of the species used in the phylogenetic analyses. Newly sequenced in the present study in bold.

	Species	Country	Subfamily	GenBank number
1	*Acrocyrtus* sp.	Thailand	Lepidocyrtinae	MT914190
2	*Ascocyrtuscinctus* Schäffer, 1898	Indonesia	Lepidocyrtinae	﻿OP094720
3	*Lepidocyrtus* sp.	Brazil	Lepidocyrtinae	MF716621
4	*Lepidocyrtusfimetarius* Gisin, 1964	China	Lepidocyrtinae	﻿MK431900
5	*Lepidocyrtusnigrosetosus* Folsom, 1927	Brazil	Lepidocyrtinae	﻿MW033192
6	*Lepidocyrtussotoi* Bellini & Godeiro, 2015	Brazil	Lepidocyrtinae	﻿MT928545
7	*Pseudosinellatumula* Wang, Chen & Christiansen, 2002	China	Lepidocyrtinae	MT611221
8	*Lepidocyrtinusdapeste* Santos & Bellini, 2018	Brazil	Seirinae	﻿MF716609
9	*Lepidocyrtinusdiamantinae* (Godeiro & Bellini, 2015)	Brazil	Seirinae	﻿MF716594
10	*Lepidocyrtinusharenus* (Godeiro & Bellini, 2014)	Brazil	Seirinae	﻿MF716617
11	*Lepidocyrtinusparaibensis* (Bellini & Zeppelini, 2009)	Brazil	Seirinae	﻿MF716600
12	*Lepidocyrtinus* ca. *prodiga* (Arlé, 1959)	Brazil	Seirinae	﻿MF716595
13	*Seiraatrolutea* (Arlé, 1939)	Brazil	Seirinae	MF716602
14	***Seiraboneti* comb. nov.**	**China**	**Seirinae**	**OP181099**
15	*Seirabrasiliana* Arlé, 1939	Brazil	Seirinae	MF716619
16	*Seirapaulae* Cipola & Bellini, 2014 (*in*: Cipola et al. 2014b)	Brazil	Seirinae	﻿MF716601
17	*Seiracoroatensis* Godeiro & Bellini, 2015	Brazil	Seirinae	MF716614
18	*Seiradowlingi* (Wray, 1953)	Brazil	Seirinae	MF716615
19	*Seiradowlingi*	China	Seirinae	﻿﻿MW419950
20	*Seiramendoncae* Bellini & Zeppelini, 2008	Brazil	Seirinae	﻿MF716597
21	*Seirapotiguara* Bellini, Fernandes & Zeppelini, 2010	Brazil	Seirinae	﻿MF716613
22	*Seiraritae* Bellini & Zeppelini, 2011	Brazil	Seirinae	MF716605
23	*Seirasanloemensis* Godeiro & Cipola, 2020	Cambodia	Seirinae	﻿﻿MT997754
24	*Seiratinguira* Cipola & Bellini, 2014 (*in*: Cipola et al. 2014b)	Brazil	Seirinae	﻿MF716620
25	*Tyrannoseirabicolorcornuta* (Bellini, Pais & Zeppelini, 2009)	Brazil	Seirinae	MF716599
26	*Tyrannoseiragladiata* Zeppelini & Lima, 2012	Brazil	Seirinae	MT914185
27	*Tyrannoseiraraptora* (Zeppelini & Bellini, 2006)	Brazil	Seirinae	MF716610

To complete our dataset, following the relationship hypothesis of Seirinae + Lepidocyrtinae (Zhang et al. 2014, 2019; Godeiro et al. 2020a, 2021), we downloaded from NCBI the 13 mitochondrial protein coding genes (PCG’s) sequences from 19 Seirinae species and seven Lepidocyrtinae to be used as outgroups. The detailed classification information and accession numbers of the 27 species analyzed in this study are listed in Table 1.

### ﻿Phylogenetic inference

Protein coding genes (PCG’s) sequences from the 27 species were translated into amino acids using TRANSDECODER v. 5.5.0 (Haas et al. 2013) and aligned separately with MAFFT (Katoh and Standley 2013), “linsi” strategy. BMGE v. 1.12 (Criscuolo and Gribaldo 2010) performed the trimming with default strategy. PHYKIT v. 1.9.0 (Steenwyk et al. 2021) was used to generate the matrices and partition schemes. Maximum Likelihood (ML) analyses were performed with IQTREE v. 2.0.7 (Minh et al. 2020), ultrafast bootstrap 1000 replicates (Hoang et al. 2018) and SH-aLRT support. Bayesian inference was performed using PHYLOBAYES-MPI v1.8 (Lartillot et al. 2013), default model CAT+GTR with four rate categories, discretized gamma distribution of rates across sites. Phylogenetic trees were visualized in FIGTREE v. 1.3.1 (Rambaut 2010).

### ﻿Morphological analysis

The type material of *Lepidocyrtinusboneti* deposited at the Muséum National d’Histoire Naturelle, France, and specimens recently collected from China (Hainan island) were analyzed. Under a stereomicroscope Teelen XTL- 207, Chinese specimens were bleached and diaphanized, first in 5% KOH and after in 10% lactophenol for 3 min/each. Hoyer’s liquid was used to mount the specimens between a slide and a glass coverslip (Christiansen and Bellinger 1980, 1998). Mounted specimens were examined using a Leica DM2500 microscope. Illustrations were made with the help of an attached drawing tube and based on photographs taken with DMC4500 camera and LEICA APPLICATION SUITE v. 4.9. Specimens in ethanol gel were photographed using a Leica stereomicroscope S8AP0 attached to a Leica DMC4500 digital sight camera. Maps of species localities were made after Shorthouse (2010). The examined material is deposited at the collections of the Shanghai Natural History Museum (**SNHM**), Shanghai, China; Invertebrate collection of the Instituto Nacional de Pesquisas da Amazônia (**INPA**); and Muséum National d’Histoire Naturelle (**MNHN**), Paris, France.

The terminology used in descriptions follows: clypeal chaetotaxy after Yoshii and Suhardjono (1992); labral chaetotaxy after Cipola et al. (2014a); labial papillae, maxillary palp and basolateral and basomedian labial fields after Fjellberg (1999), but using the Gisin’s system (1964) for naming the chaetae rows; postlabial chaetotaxy after Chen and Christiansen (1993) and Cipola et al. (2018a); subcoxae outer chaetotaxy after Yosii (1959); trochanteral organ after Christiansen (1958b) and South (1961); unguiculus lamellae after Hüther (1986); male genital plate after Christiansen (1958a); and manubrial ventral formula after Christiansen and Bellinger (2000). The head dorsal chaetotaxy was described based on Mari-Mutt (1979) and that of the body based on Szeptycki (1979), both with additions of Soto-Adames (2008), Cipola et al. (2018a), and Zhang et al. (2019); and specialized chaetae (S-chaetae) after Zhang and Deharveng (2015). Symbols used to depict the chaetotaxy are presented in Fig. 3. Chaetotaxy data are all given by one side of body only, except for the head plate.

### ﻿Abbreviations used in the description

**Abd** abdominal segment(s);

**ae** antero-external lamella;

**ai** antero-internal lamella;

**Ant** antennal segment(s);

**a.t.** unpaired apical tooth;

**b.c.** basal chaeta of maxillary palp;

**b.t.** paired basal teeth;

**f** frontal chaetae of clypeus;

**l** lateral chaetae of clypeus;

**l.p.** lateral process of papilla E;

**mac** macrochaeta(e);

**m.t.** unpaired median tooth;

**ms** specialized microchaeta(e);

**pf** prefrontal chaetae of clypeus;

**psp** pseudopore(s);

**pe** postero-external lamella;

**pi** postero-internal lamella;

**sens** specialised ordinary chaeta(e);

**t.a.** terminal appendage of maxillary palp;

**Th.** thoracic segment(s).

## ﻿Results

### ﻿Mitogenome features and phylogenetic placement

**Figure 1.**
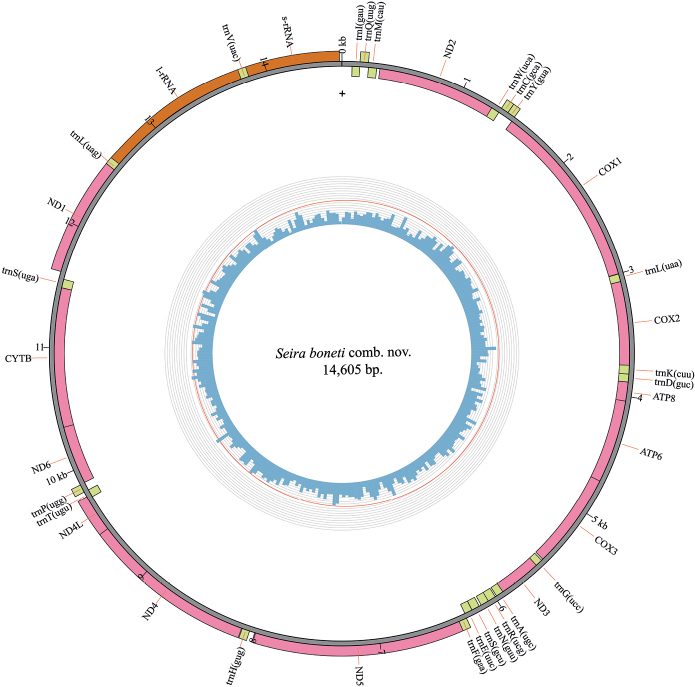
Circular representation of the mitogenome of *Seiraboneti* comb. nov. The innermost circle shows the GC content and the outermost circle shows the gene features, rRNAs (orange), tRNAs (green), and PCGs (pink), (+) indicates the side of the major J-strand.

The assembled mitogenome of *Seiraboneti* comb. nov. is a circular molecule of 14,605 bp (Fig. 1). All 13 protein coding genes, 22 tRNA, and two rRNA were successfully recovered. The most frequent gene order observed in springtails, the same as the Pancrustacea gene order, was observed in the mitogenome of *S.boneti* comb. nov. The genome base composition was as follows: A (38%, 5587), T (35%, 4939), G (10%, 1512), C (17%, 2567).

**Figure 2.**
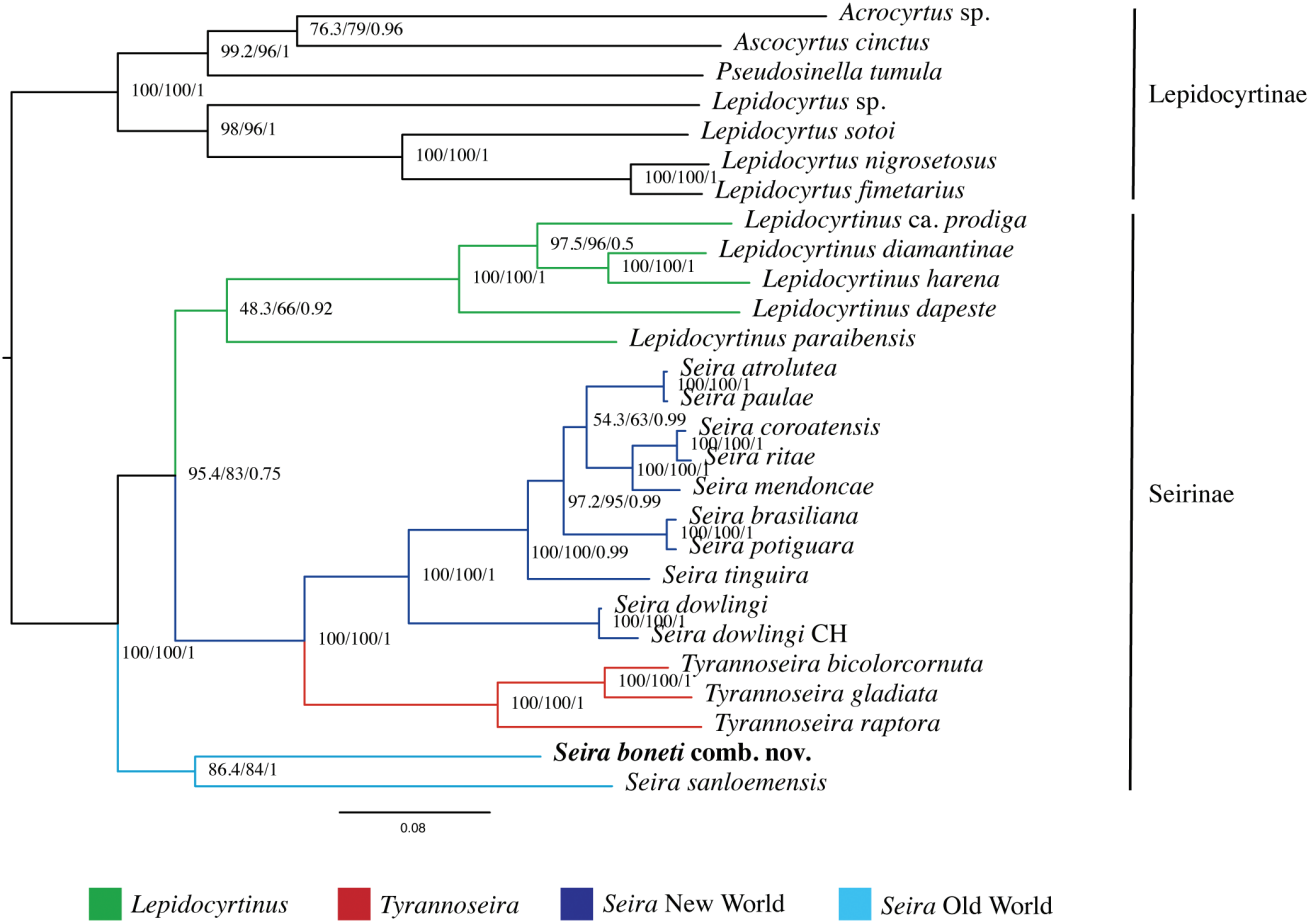
Phylogenetic placement of *Seiraboneti* comb. nov. (in bold) based on Bayesian Inference and Maximum Likelihood analyses. Numbers in the nodes represent the SH-aLRT support, bootstrap values (both for Maximum Likelihood), and the posterior probability (Bayesian Inference support), respectively.

The final matrix containing the 13 PCG’s concatenated of the 27 species had a length of 3,391 amino acid sites. Maximum likelihood and Bayesian inference analyses placed *Seiraboneti* comb. nov. in the same branch as *Seirasanloemensis* from Cambodia with moderate support values (86.4/84/1 - SH-aLRT support, bootstrap, and posterior probability, respectively). Also, the monophyly of Seirinae was recovered with high support values (100/100/1) (Fig. 2). Regarding the Neotropical species, the phylogeny remained the same as previous results (Godeiro et al. 2020a, b, 2021; Godeiro and Zhang 2021). This result corroborates our previous finding that the Asian population is likely to belong to a different lineage of Seirinae.

### ﻿Taxonomy

**Class Collembola Lubbock, 1873**

**Order Entomobryomorpha Börner, 1913**

**Family Entomobryidae Tömösváry, 1882**

**Subfamily Seirinae Yosii, 1961 (in 1961b) sensu Zhang et al., 2019**

#### Genus *Seira* Lubbock, 1870

##### ﻿ Seira boneti

Taxon classificationAnimaliaCollembolaEntomobryidae

(Denis, 1948) comb. nov.

F8D28BDE-CD86-55FC-B437-4FED5157AA55

**Figure 3.**
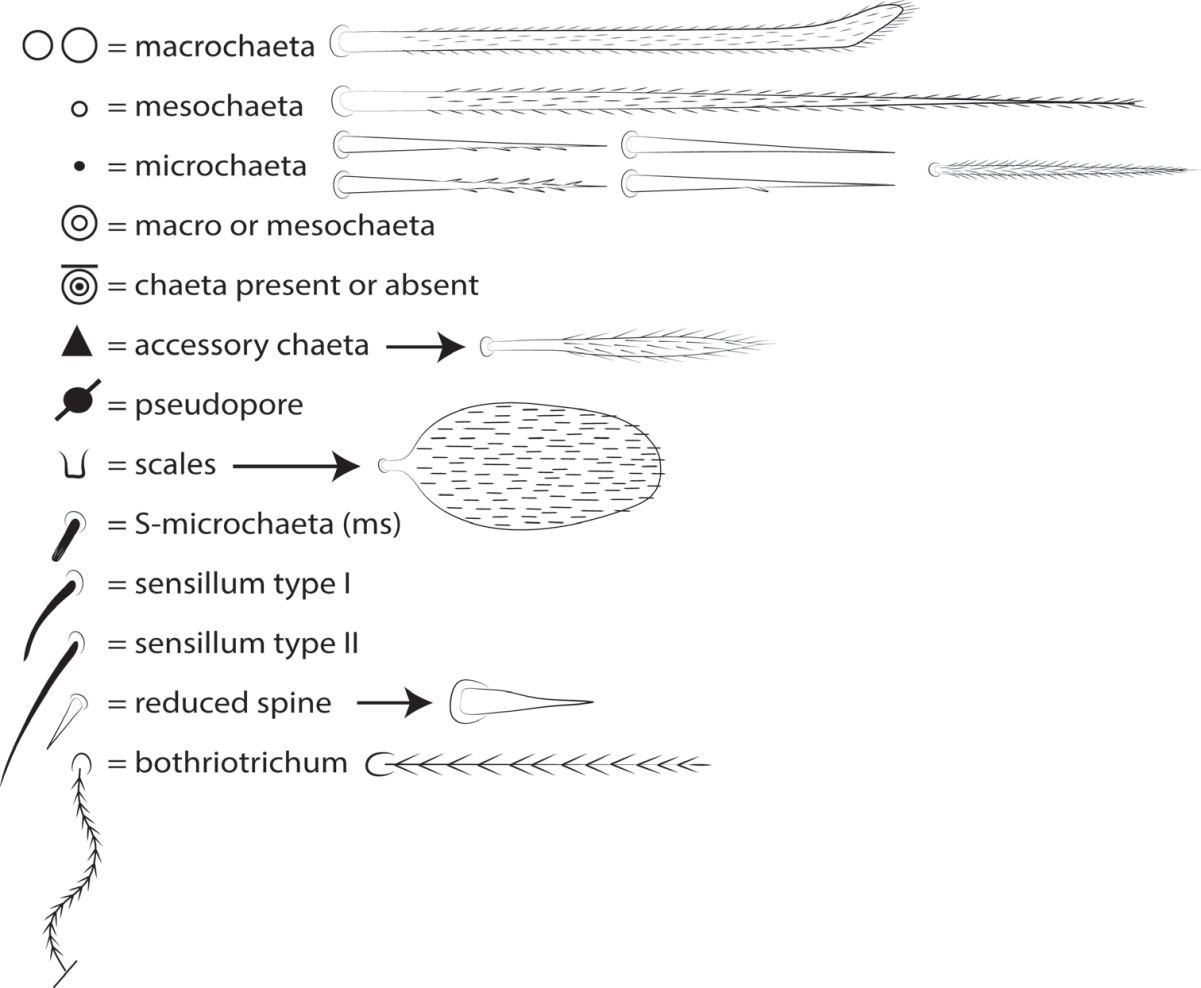
Symbols used in the chaetotaxy redescription of *Seiraboneti* comb. nov.

**Figure 4.**
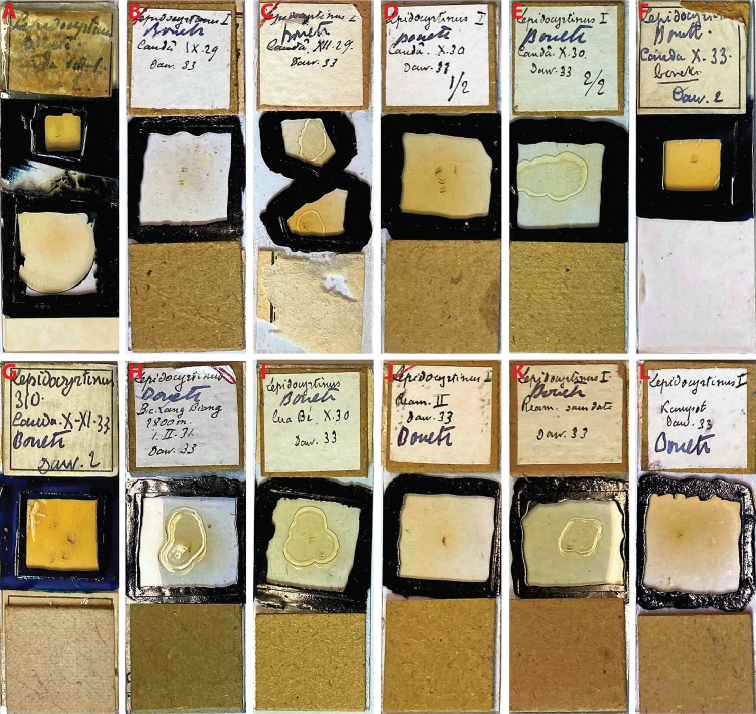
Syntypes slides of *S.boneti* comb. nov. deposited in MNHN (Paris, France) **A–H** specimens from the type locality herein designated, Câuda [Cầu Đá, Nha Trang], Vietnam **A** lectotype designed **B–H** paralectotypes herein designated **I** specimens from Pic Lang Biang [Lang Biang Peak, Dalat], Vietnam **J** specimen from Cua-Be [Cầa Bé, Nha Trang], Vietnam **K** specimens from Réam [Ream], Cambodia **L** specimen from Kampot, Cambodia.

**Figure 5.**
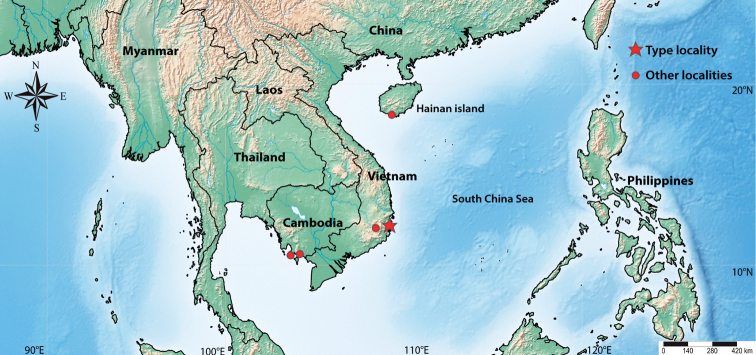
Records map of *S.boneti* comb. nov. in Southeast Asia; star represents the type locality herein designated, Cầu Đá (Vietnam); circles in Cambodia and Vietnam are additional localities reported in the original description (Denis, 1948), and circle in Hainan Island is a new record for China.

**Figure 6.**
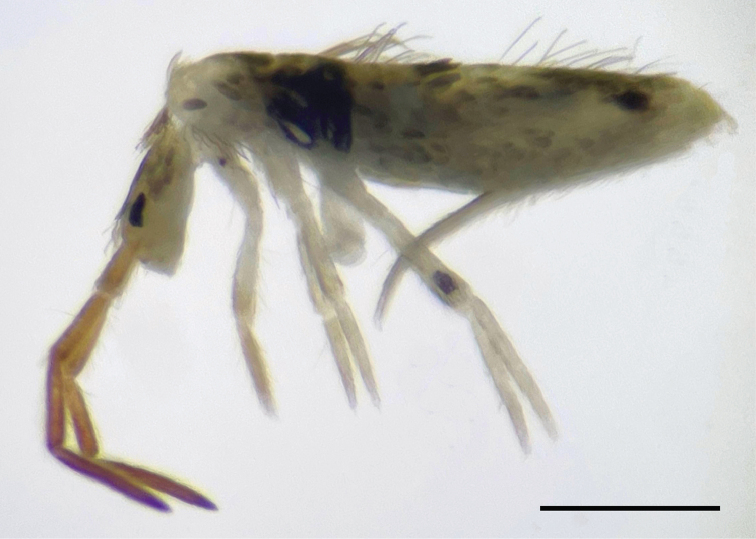
*Seiraboneti* comb. nov.: habitus of specimen fixed in alcohol from Hainan Island, China (lateral view). Scale bar: 0.5 mm.

**Figure 7.**
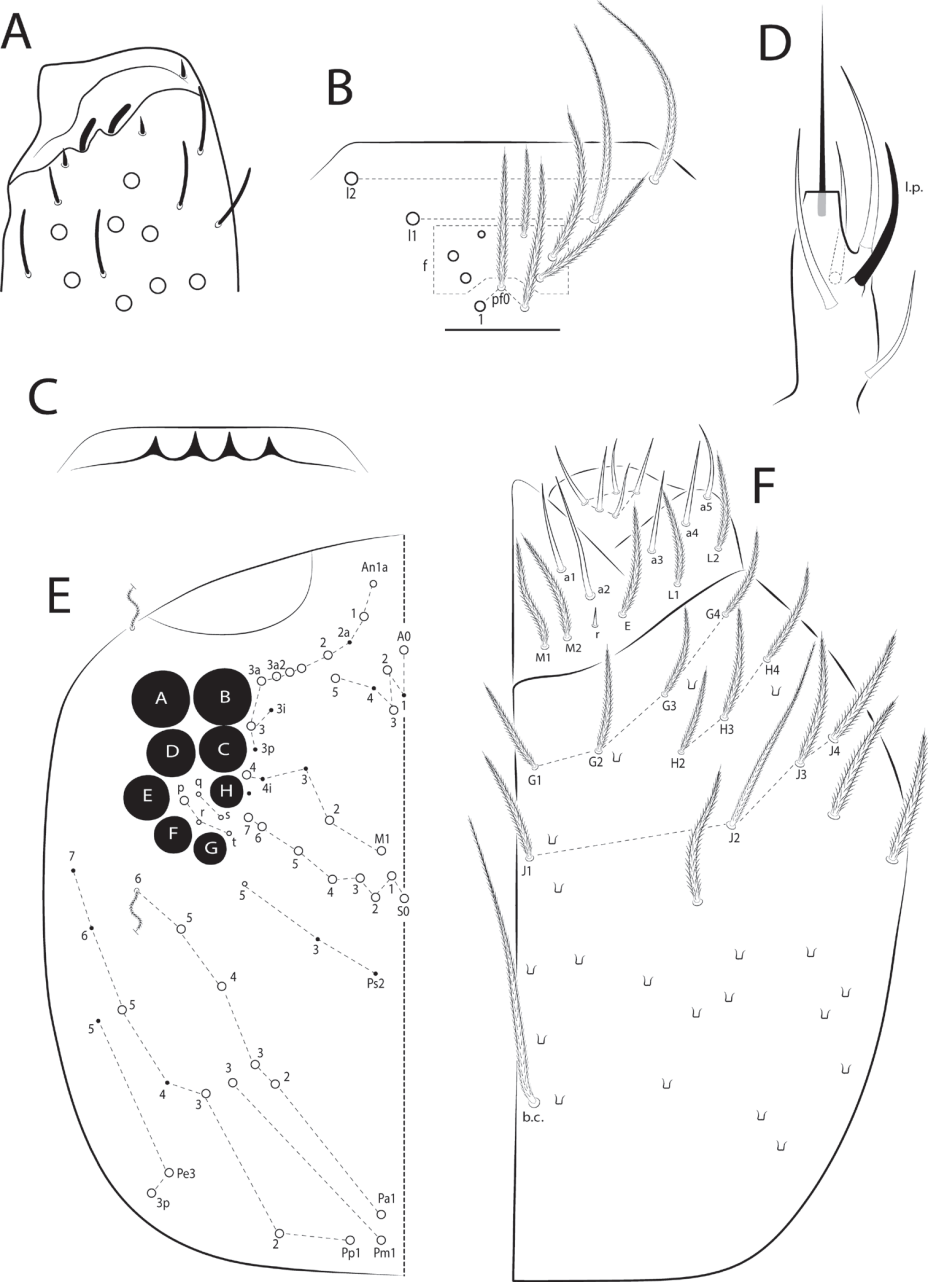
*Seiraboneti* comb. nov.: head **A**Ant III distal chaetotaxy (lateral view) **B** clypeal chaetotaxy **C** labral papillae **D** labial papilla E (right side) **E** head dorsal chaetotaxy (left side) **F** labial proximal chaetae, basomedian and basolateral labial fields, and complete postlabial chaetotaxy (right side).

**Figure 8.**
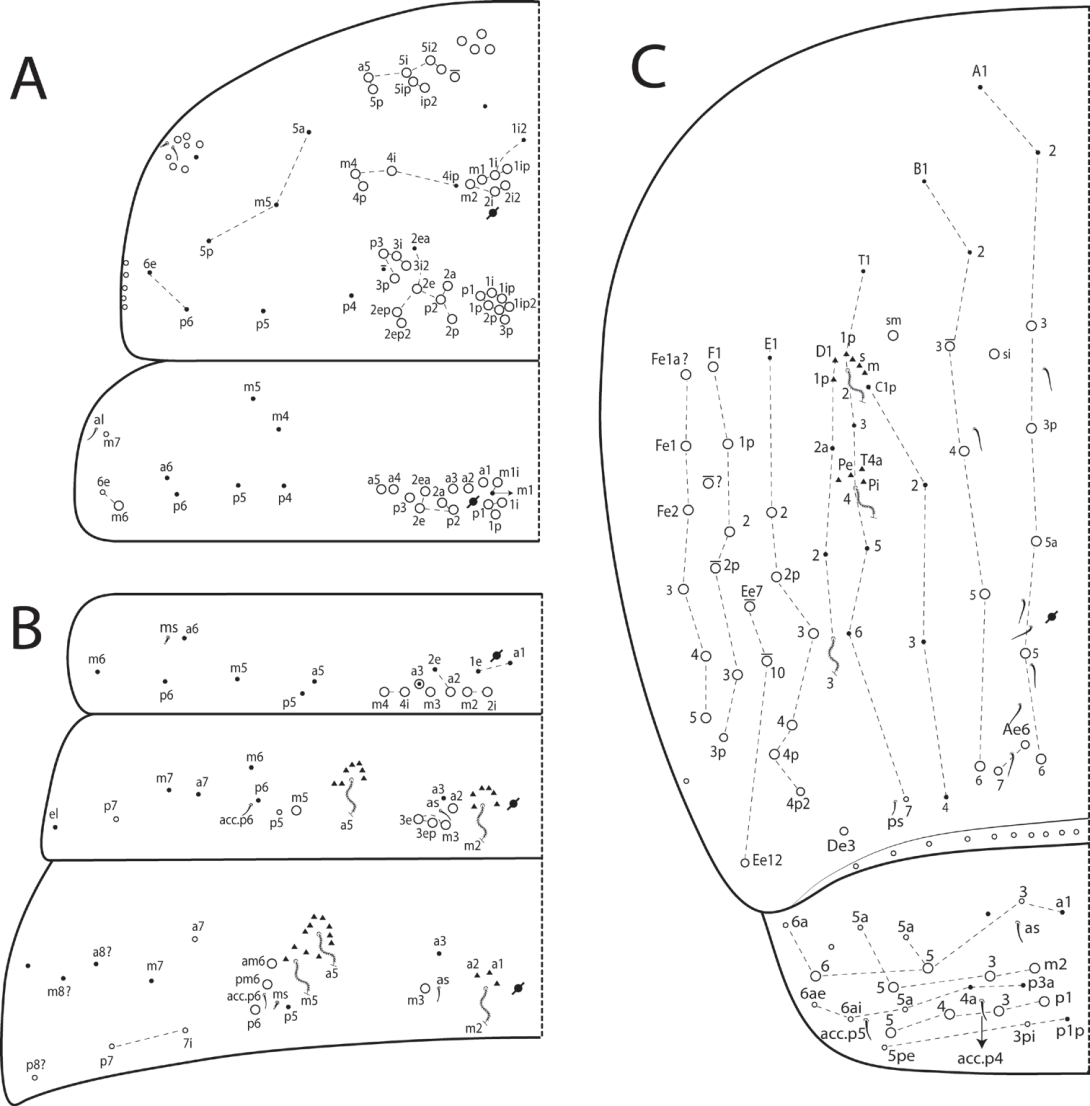
*Seiraboneti* comb. nov.: dorsal chaetotaxy (left side) **A**Th II–III **B**Abd I–III **C**Abd IV–V.

**Figure 9.**
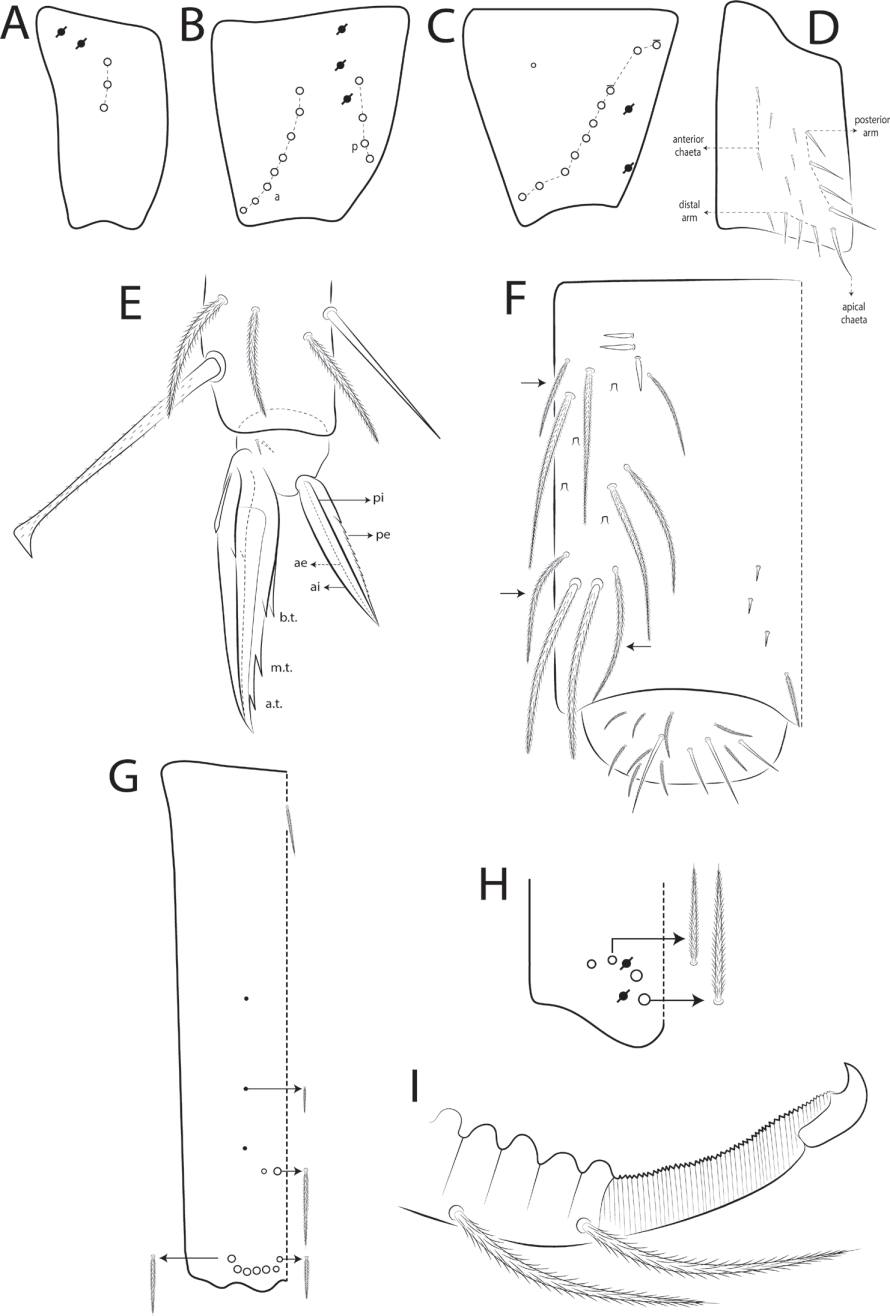
*Seiraboneti* comb. nov.: trunk appendages **A–C** chaetotaxy of subcoxa I–III, respectively (outer side) **D** trochanteral organ (posterior side) **E** distal tibiotarsus and empodial complex III (posterior view) **F** collophore (lateral view), arrows in the anterior side indicate chaetae present or absent **G** manubrium ventral chaetotaxy **H** manubrial plate (dorsal view) **I** distal dens and mucro (outer view).

Figs 3 , 4 , 5 , 6 , 7 , 8 , 9

Lepidocyrtinus boneti Denis, 1948: 261, fig. 26, Vietnam and Cambodia.

###### Typological note.

*Seiraboneti* comb. nov. was described based on 18 specimens from five localities of Vietnam and Cambodia (Figs 4, 5), which are deposited in the MNHN, Paris. The description of *S.boneti* comb. nov. was based on a series of 13 syntypes (ICZN 2000, chapter 16, Article 73.2), without a designated holotype. Hence, we designate a lectotype, as well as paralectotypes for the other specimens from the same locality, Câuda [Cầu Đá, Nha Trang], Vietnam (ICZN 2000, chapter 16, Articles 73.2.2 and 74). From now, this becomes the type locality of *S.boneti* comb. nov. (ICZN 2000, chapter 16, Articles 73.2.3 and 76.2).

###### Type material.

***Lectotype*** (sex unclear, not possible to see) designated on slide (MNHN): Vietnam, Câuda [Cầu Đá, Nha Trang], viii–i (Figs 4A, 5). ***Paralectotypes*** designated on slides (MNHN): three specimens with the same data as the lectotype, two specimens collected ix.1929, one collected xii.1929, four collected x.1930, one collected x.1933, and one collected x-xi.1933 (Fig. 4B–G).

###### Other examined material.

One specimen in slide (MNHN): Vietnam, Pic Lang Biang [Lang Biang Peak, Dalat], 2,100 m., 1.ii.1931. One specimen on slide (MNHN): Vietnam, Cua-Be [Cầa Bé, Nha Trang], x.1930. Two specimens pn slides (MNHN): Cambodia, Réam [Ream]. One specimen in slide (MNHN): Cambodia, Kampot (Figs 4H–L, 5). One male and four females on slides (SAN4 1–5): China, Hainan Island, Sanya Beach, Dadonghai forest in litter samples collected near ancient tombs, 18°13'06.8"N, 109°30'14.6"E (Fig. 5), rain forest, 20 meters of altitude, entomological aspirator, 05.x.2021, NN Godeiro leg.

###### Diagnosis.

Body with dark lateral spots on Th III–Abd I (rarely absent) and 1+1 smaller one on Abd IV posteriorly. Ant IV not annulated with an apical bulb bilobed; labral papillae conical, outer slightly smaller; labial papilla E l.p. apically thinner and exceeding the apex of the papilla; head macrochaetotaxy with 8 ‘An’, 4 ‘A’, 3 ‘M’, 8 ‘S’, 5 ‘Pa’, 2 ‘Pm’, 4 ‘Pp’ and 2 ‘Pe’ mac; Th II with 11–12 anterior, 9 median and 17 posterior mac (p5 mac absent); Th III–Abd III with 14, 6–7, 4 and 1 inner mac, respectively; Abd IV with 12 or 13 inner and 19–23 lateral mac; trochanteral organ with about 16–18 spine-like chaetae; unguis a.t. present and unguiculus pe lamella serrated; collophore anteriorly with 2 distal mac, posteriorly with 3 spines on each side, lateral flap with 4 smooth and 9 ciliated chaetae; manubrium ventrally with 4 subapical chaetae, outer chaeta smaller than the inner chaeta; manubrial plate with 4 chaetae.

###### Note.

On the basis of color pattern and morphological information extracted from the lectotype and type material (despite the poor state of conservation), we consider that both populations are conspecific.

###### Redescription.

***Body*.** Total length (head + trunk) of specimens 2.28–2.51 mm (*n* = 2 paralectotypes), lectotype 1.68 mm. Specimens whitish with brownish pigment on Ant I–IV; dark blue pigment forming a spot on Th III–Abd I laterally (rarely absent) and a smaller spot postero-laterally on Abd IV, coxae I, and femur III distally pigmented in dark-blue; eyepatches black (Fig. 6). Scales heavily ciliated, oval or elongated and apically rounded (rarely truncate, pointed or irregular) present on Ant I to proximal 1/4 of Ant III, dorsal and ventral head, thorax, and abdomen, legs (except empodia), collophore anteriorly, both sides of the manubrium and dens ventrally; mac heavily ciliated apically, slightly foot shaped, rounded or acuminate; smooth microchaetae apically ramified or simple (Fig. 3).

***Head*.** Antennae shorter than the trunk (Fig. 6); ratio antennae: trunk = 1: 1.46–1.68 (n=3), lectotype 1: 1.46; antennal segments ratio as I: II: III: IV = 1: 1.24–1.55: 1.33–1.75: 2.20–3.11, lectotype 1: 1.55: 1.55: 2.75. Ant IV not annulated, with an apical bulb bilobed. Ant III distally with 2 finger-shaped sens (apical organ), some sens of different sizes and ciliated chaetae (Fig. 7A). Ant I dorsally with 1 outer mac, 2 median sens, and 3 proximal sens-like smooth chaetae. Clypeal formula with 4 (l1–2), 6 (f), 3 (pf0–1) ciliated chaetae, l1–2 larger than the others and apically acuminate, two f smaller, others subequal (Fig. 7B). Four prelabral ciliated chaetae; labral formula with 4 (a1–2), 5 (m0–2), 5 (p0–2) smooth chaetae; a1 regular (not thick), p0–1 larger than others. Four labral papillae conical, outer papillae slightly smaller than the inner papillae (Fig. 7C). Labial palp with five main papillae (A–E) plus one hypostomal papilla (H) with 0, 5, 0, 4, 4, 2 guard appendages, respectively; labial papilla E with l.p. apically thinner and exceeding the apex of the papilla (Fig. 7D). Eyes 8+8, A and B larger than the others, G and H smaller, with 5 interocular chaetae (q, s, p, r, t); head dorsal chaetotaxy (Fig. 7E) with 8 ‘An’ (An1a–1, An2–3), 4 ‘A’ (A0, A2–3, A5) , 3 ‘M’ (M1–2, M4), 6 ‘S’ (S0–7), 5 ‘Pa’ (Pa1–5), 2 ‘Pm’ (Pm1, Pm3), 4 ‘Pp’ (Pp1–4, Pp5) and 2 ‘Pe’ (Pe3–3p) mac; 2 pairs of bothriotricha (1 subantennal and 1 post-ocellar) present. Basolateral and basomedian labial fields with chaetae a1–5 smooth (a2 largest), M1–2, E, L1–2 ciliated, r in a small smooth spine; labium with 5 subequal smooth chaetae (Fig. 7F). Maxillary palp with t.a. smooth and b.c. rough, thicker and 1.15 larger than the t.a.; sublobal plate internally with 3 smooth main appendages (1 proximal slightly thinner and shorter) plus a minute smooth appendage distally. Ventral chaetotaxy with 15 ciliated chaetae; postlabial chaetotaxy with 4 (G1–4), 3 (H2–4), 4 (J1–4) chaetae, b.c. and J2 thin, acuminate and elongated (J2 smaller that b.c.), others subequal (Fig. 7F).

***Thorax chaetotaxy*** (Fig. 8A). Th II ‘a’, ‘m’ and ‘p’ series with 11–12 (group a5, excluding the anterior collar), 9 (m1–1ip, m2–2i2, m4i–4p) and 17 (p1ip2–p1p3, p2a–2p, p2e–2ep, p3p–3i2) mac, respectively. Th III ‘a’, ‘m’ and ‘p’ series with 5 (a1–5), 2 (m1i, m6) and 8 (p1i–1p, p2a–2ea, p3) mac, respectively. Th II–Abd V with ms and sens formula 1, 0| 1, 0, 1, 0, 0 and 1, 1| 0, 2, 2, 8, 3, respectively (Fig. 8). Ratio Th II: III = 1.67–1.45: 1 (*n* = 3), lectotype 1.67: 1.

***Abdomen chaetotaxy*** (Fig. 8B, C). Abd I ‘a’, ‘m’ and ‘p’ series with 1–2 (a2–3), 5 (m2i–2, m3, m4–4i) and 0 mac, respectively. Abd II ‘a’, ‘m’ and ‘p’ series with 1 (a2), 4 (m2–3e, m5), 0 mac, respectively; a5 and m2 as bothriotricha with 7 and 5 accessory chaetae, respectively. Abd III ‘a’, ‘m’ and ‘p’ series with 0, 3 (m3, am6, pm6) and 1 (p6) mac, respectively; m2 bothriotrichum with 3 accessory chaetae (a1–2 and 1 unnamed), and bothriotricha a5 and m5 with 11 accessory chaetae between them. Abd IV with 12 or 13 inner mac on A–T series (A3–3p, A5a–6, Ae6–7, B3–6, si, sm) and 19–23 lateral mac on D–Fe series (De3, E2–4p2, Ee7, Ee10, Ee12, F1–3p, one of unknown homology, Fe1a?–5); at least 8 sens (ps type I, others type II) and 10 posterior mesochaetae. Abd V ‘a’, ‘m’ and ‘p’ series with 2 (a5–6), 3 (m2–3, m5) and 4 (p1, p3–5) mac, respectively. Ratio Abd III: IV = 1: 3.33–4.10 (n=3), lectotype 1: 4.10. Abd II–IV bothriotrichal formula 2 (a5, m2), 3 (a5, m2, m5), 3 (T2, T4, D3) (Fig. 8B, C).

***Legs*.** Subcoxa I with one row of 3 chaetae and 2 psp; subcoxa II with an ‘a’ row of 8 chaetae, ‘p’ row of 4 chaetae and 3 psp; subcoxa III with one row of 8–10 chaetae, 1 anterior chaeta and 2 posterior psp (Fig. 9A–C). Trochanteral organ with 16–18 spine-like chaetae, at least 2 anterior, 4 posterior, 5 internal, 1 apical and 4 distal arms (Fig. 9D). Anterior side of femurs II and III with 1 small proximal spine-like chaeta. Tibiotarsus outer side distally with 1 tenent hair ciliated, apically capitate, and subequal to unguis outer length; inner side of tibiotarsus III with 1 smooth chaeta slightly longer than the unguiculus. Pretarsus with 1 minute anterior and 1minute posterior smooth chaetae (Fig. 9E). Unguis outer side with a pair of lateral teeth and 1 unpaired median tooth; inner side with 4 teeth, b.t. on proximal half, m.t. on distal one fourth and slightly longer than b.t., a.t. on distal one eighth and subequal to b.t. Unguiculus with all lamellae acuminate and smooth (ai, ae, pi, pe), except pe serrate and with a minute tooth on distal half (Fig. 9E).

***Collophore*** (Fig. 9F). Anterior side with ~ 10–13 chaetae, 3 proximal smooth spine-like chaetae, 3 median ciliated mac thin and apically acuminate, 1 thin and 1–4 regular ciliated chaetae, and distally 2 ciliated mac; posterior side with 3 subapical spines and 1 apical ciliated chaeta; lateral flap with about 13–14 chaetae, 4 smooth (2 larger than the others) and 9 ciliate.

***Furcula*.** Manubrium ventral chaetotaxy with 1, 2, 2, 2/4 (subapical), 14 (apical) ciliated chaetae, outer subapical chaetae smaller than the inner chaetae (Fig. 9G). Manubrial plate with 2 psp and 4 ciliated chaetae, the 2 inner chaetae larger than the lateral ones (Fig. 9H). Mucro falcate (only the distal tooth apparent) and without basal spine; proximal tooth reduced and enveloped by the dens cuticle (Fig. 9I).

###### Remarks.

The present study increased substantially the morphological detailing of *Seiraboneti* comb. nov. compared to the original description. Considering that most species of *Seira* from Asia are also poorly described (Denis 1948; Yoshii and Suhardjono 1992; Nguyen 2001; Cipola et al. 2018a), we compared *S.boneti* comb. nov. with other Asian species with 7 mac on Abd I (*S.cinerea* Yosii, 1966, *S.nidarensis* Baquero & Jordana, 2014, *S.simbalwaraii* Baquero & Jordana, 2015 from India, and *S.urbana* Nguyen, 2001 from Vietnam) or 6 mac (*S.hazrai* Baquero & Jordana, 2014 and *S.prabhooi* Baquero & Jordana, 2015 from India) (Table 2). These species also resemble each other in dense head macrochaetotaxy (except for *S.cinerea* and *S.urbana*, which their head chaetotaxy are unknown), although *S.nidarensis* and *S.hazrai* differ from the others by the presence of Ps2 mac. They also resemble each other in Th II by PmA–B groups respectively with 6 or 7 and 3 mac (but differ from each other in PmC group), Th III with 13–14 inner mac apparently with the same homology (a1–5, m1i, p1i–1p, p2a–p2ea, p3), and Abd II–III respectively with 4 (a2, m3–3e, 3ep) and 1 (m3) inner mac, although this last segment was not described in *S.cinerea*. *Seiraboneti* comb. nov. is more similar to *S.urbana* in body color pattern with a lateral spot on Th II–Abd II and another on And IV posteriorly, Th II anteriorly with some extra mac, and prelabral and labral chaetotaxy (see Nguyen 2001). Due to the similarities between these two species which coexist in the same region (Indochina), there is a risk they are synonyms. For this reason, we tried to consult the type material of *S.urbana* deposited at Vietnam Academy of Science and Technology, Vietnam, but the loan was not provided by the responsible (Dr. Anh T. T. Nguyen). Although the material was not obtained for a more rigorous comparison, based on the literature *S.boneti* comb. nov. differs from *S.urbana* in Th II anteriorly with 11–12 mac (8 in *S.urbana*), Abd IV with 12–13 inner mac (11 in *S.urbana*), unguiculus pe lamella serrated (apparently smooth in *S.urbana*), and collophore anteriorly with 3 proximal spine-like chaetae (4 in *S.urbana*).

**Table 2.** Comparison between *Seira* species from Asia with 6–7 and 4 central macrochaetae on Abd I–II, respectively.

		Species						
		*S.boneti* comb. nov.	*S.cinerea*	*S.nidarensis*	*S.simbalwaraii*	*S.urbana*	*S.hazrai*	*S.prabhooi*
	References:	(1–2)	(3)	(4)	(5)	(6)	(4)	(5)
	Type locality:	Vietnam and Cambodia	Bombay, Afghanistan	Himalayas, India	H. Pradesh, India	Hanoi, Vietnam	Himalayas, India	H. Pradesh, India
**Characteristics**								
Head pigments		–	laterally	dorsally	anteriorly	–	posteriorly (+/–)	except dorsal part
Trunk stains pattern		lateral spot on Th II–Abd I	transversal band on Th II–Abd III	all Th II–Abd III	transversal band on Th II–Abd III	lateral spot on Th III–Abd II	All Th II–Abd III or only Abd II–III	–
Abd IV pigments		spots latero-posterior	band laterally	irregular spots	1/3 posteriorly	spots latero-posterior	central spot (+/–)	spot anteriorly (+/–)
Ant IV apical bulb		2	?	1	1	?	1	1–2
Ant IV annulated		–	–	+	–	+	–	–
Head mac	**Sutural**	8	?	7	5	?	7	5
	**Ps2**	–	?	+	–	?	+	–
	**Pa4**	+	?	+	+	?	+	+
Th II mac	**m1–2** complex	6	?	4	6	6	4	5
	**PmA** group	7	7	6	7	7	6	7
	**PmC** group	7	7	5	9	9	5	7
	**p5 mac**	–	–	+	–	–	+	–
Th III central mac		14	13	14	14	14	14	13
Abd I mac		6–7	7	7	7	7	6	6
Abd IV central mac		12–13	?	11	14	11	13	11
Trochanteral organ		16–18	25	11–13	25	20–25	7	11
Manubrial plate chaetae		4	?	4–5	5	?	4	?

Abbreviations: (+) present; (–) absent; (?) unknown. References: ^(1)^this study; ^(2)^Denis 1948; ^(3)^Yosii 1966; ^(4)^Baquero et al. 2014; ^(5)^Baquero et al. 2015; ^(6)^Nguyen 2001.

## ﻿Discussion

Whereas the Asian continent currently has almost 25% (~ 50 spp.) of the known richness of Seirinae (Bellinger et al. 1996–2023), the moderate support values clustering *S.boneti* comb. nov. and *S.sanloemensis* are possibly related to the absence of other Asian taxa in the present phylogenetic analysis, mainly species morphologically similar to them, such as the six compared above with *S.boneti* comb. nov. (Yosii 1966; Nguyen 2001; Baquero et al. 2014, 2015). This makes sense considering that phylogenetically related the Neotropical *Seira* species (e.g. *S.atrolutea* Arlé, 1939, *S.paulae* Cipola & Bellini, 2014 (*in*: Cipola et al. 2014b), *S.coroatensis* Godeiro & Bellini, 2015, *S.ritae* Bellini & Zeppelini, 2011, and *S.mendoncae* Bellini & Zeppelini, 2008) are morphologically similar in the pattern of dorsal chaetotaxy, such as head with at least 6 central mac (M1–2, S0–3) and 11 posterior mac (Pa1–5, Pm1–3, Pp1–3, Pp5) and Abd I with 5 mac (m2i–4) (Bellini and Zeppelini 2008, 2011; Cipola et al. 2014b; Godeiro and Bellini 2015). Such observation supports that this pattern of chaetotaxy on head and Abd I (Figs 7E, 8B) likely arose at least twice within Seirinae, once within derived Neotropical *Seira* species, and once within *Seira* from the Oriental region (e.g. *S.boneti* comb. nov. and *S.sanloemensis*) and in *Lepidocyrtinus* (Fig. 2). Consequently, if such a hypothesis is confirmed by further phylogenies, it may also indicate that there was a gradual gain of body macrochaetae in the Neotropical species of *Seira*, but the evolution of these characters is still unknown for Old World species.

From the recovered topology it is also possible to infer the evolution of other characters among the Seirinae. In *Lepidocyrtinus*, the developed lateral tooth on the unguis is likely a synapomorphy of the genus among the Seirinae, but it is not exclusive, as it is also present in other genera of Entomobryinae, such *Acanthocyrtus* Handschin, 1925, *Amazhomidia* Cipola & Bellini, 2016 (in Cipola et al. 2016), *Epimetrura* Schött, 1925 and *Lepidocyrtoides* Schött, 1917, suggesting the structure emerged more than once within Entomobryidae (Schött 1925; Cipola et al. 2016, 2017, 2018b; Cipola and Greenslade 2022). Still in *Lepidocyrtinus*, modified macrochaetae on dens represent an autapomorphy which appeared in the most derived taxa, as this characteristic is absent in the basal groups, like *L.paraibensis* (Bellini and Zeppelini 2009) (Fig. 2).

Molecular evidence justifies the transfer of *S.boneti* comb. nov. to *Seira* as found in the present study, clustering the species with another congener, *S.sanloemensis*. Such topology was also recovered in Bellini et al. (2023), although in this latter study the authors considered *L.boneti* as a *Seira* species based on preliminary data of the present study. Contrarily, the use of “*Seiraboneti*” in the online database of Bellinger et al. (1996–2023) was possibly following the outdated classification of Yosii (1959, 1961a), in which *Lepidocyrtinus* was considered as a subgenus of *Seira*. This classification was updated in Godeiro et al. (2020a) based on species from the Neotropical region, which raised *Lepidocyrtinus* to the genus level again. Although many species in Bellinger et al. (1996–2023) were transferred back to *Lepidocyrtinus* following this new classification, this was not the case of *L.boneti*.

In addition to the molecular evidence, morphologically this transfer can also be explained by the presence of head posterior macrochaetae (usually absent in *Lepidocyrtinus*), mesothorax normal (usually projected anteriorly in *Lepidocyrtinus*), and the absence of developed lateral teeth in the unguis (Fig. 9E), which is exclusive to *Lepidocyrtinus* compared with other Seirinae (see Cipola et al. 2020).

Our results corroborated that *S.boneti* comb. nov. belongs to an Old World *Seira* lineage (Fig. 2), although to reveal the natural groups and possibly to update new classifications, it is necessary to include more species from other continents, mainly European taxa of the *Seiradomestica* group (Nicolet, 1842) (e.g., Cipola et al. 2018a), which is the type species of the genus. At this point, looking only to our phylogenetic tree, *Seira* needs to be better evaluated in the future, because the genus is either a non-monophyletic group, or part of its taxa are classified improperly, and consequently it would be necessary to split the genus.

## ﻿Conclusion

The present study redescribes *Seiraboneti* comb. nov. Also, based on analyses including its mitogenome and 26 other sequences of Entomobryidae species, we surveyed its phylogenetic placement. This study is part of an on-going biogeographical and evolutionary study of the route of Seirinae global dispersion. To comprehend the evolutionary history of the subfamily we need comprehensive worldwide sampling and sufficient molecular markers. Available evidence suggests that the subfamily could be reorganized based on molecular data, especially given that our preliminary results grouped the two sampled Asian species into a distinct, ancestral clade relative to all New Word species of Seirinae.

## Supplementary Material

XML Treatment for Seira boneti

## References

[B1] Arlé R (1939) Collembola. Anexo n. 2 ao relatório da excursão científica do Instituto Oswaldo Cruz realizada na zona da E. F. N. O. B., em outubro de 1938. Boletim Biológico. Nova Série 4(2): 295–300.

[B2] Baquero E Mandal G Jordana R (2014) Singular Fauna of Entomobryidae (Collembola) from “Land of Passes” in the Himalayas, India. The Florida Entomologist 97(4): 1554–1587. 10.1653/024.097.0430

[B3] Baquero E Mandal G Jordana R (2015) Entomobryoidea (Collembola) from Himachal Pradesh (India) in the Himalayas. Zootaxa 4027(1): 1–41. 10.11646/zootaxa.4027.1.1 26624165

[B4] Bellinger PF Christiansen KA Janssens F (1996–2023) Checklist of the Collembola of the World. http://www.collembola.org [Accessed 25 January 2023]

[B5] Bellini BC Zeppelini D (2008) A new species of *Seira* (Collembola: Entomobryidae) from northeastern Brazil. Revista Brasileira de Zoologia 25(4): 724–727. 10.1590/S0101-81752008000400018

[B6] Bellini BC Zeppelini D (2011) A new species of *Seira* (Collembola, Entomobryidae, Seirini) from Northeastern Brazilian coastal region. Revista Brasileira de Zoologia 28(3): 403–406. 10.1590/S1984-46702011000300015

[B7] Bellini BC Fernandes LH Zeppelini D (2010) Two new species of *Seira* (Collembola, Entomobryidae) from Brazilian coast. Zootaxa 2448(1): 53–60. 10.11646/zootaxa.2448.1.4

[B8] Bellini BC Zhang F Souza PGC Santos-Costa RC Medeiros GS Godeiro NN (2023) The Evolution of Collembola Higher Taxa (Arthropoda, Hexapoda) Based on Mitogenome Data. Diversity (Basel) 15(1): 7. 10.3390/d15010007

[B9] Börner C (1903) Neue altweltliche Collembolen nebst Bemerkungen zur systematik der Isotominen und Entomobryinen. Sitzberg. Gesellshaft Naturforschender Freunde Berlin 3: 129–182. 10.5962/bhl.part.29866

[B10] Börner C (1913) Die Familien der Collembolen. Zoologischer Anzeiger 41: 315–322.

[B11] Chen JX Christiansen KA (1993) The genus *Sinella* with special reference to *Sinella* s. s. (Collembola: Entomobryidae) of China. Oriental Insects 27(1): 1–54. 10.1080/00305316.1993.10432236

[B12] Christiansen K (1958a) The Entomobryiform male Genital Plate. Proceedings of the Iowa Academy of Science 65: 474–476.

[B13] Christiansen K (1958b) The Nearctic members of the genus *Entomobrya* (Collembola). Bulletin of the Museum of Comparative Zoology 118(7): 440–545.

[B14] Christiansen K Bellinger P (1980) The Collembola of North America, North of the Rio Grande: A taxonomic analysis. Grinnell College, Iowa, 1518 pp.

[B15] Christiansen K Bellinger P (1998) The Collembola of North America, North of the Rio Grande: A taxonomic analysis. 2^nd^ Edition. Grinnell College, Iowa, 1520 pp.

[B16] Christiansen K Bellinger P (2000) A survey of the genus *Seira* (Collembola: Entomobryidae) in the Americas. Caribbean Journal of Science 36: 39–75.

[B17] Cipola NG Greenslade P (2022) Two new species of *Acanthocyrtus* Handschin, 1925 (Collembola, Entomobryidae, Entomobryinae) from Western Australia. Zootaxa 5124(3): 341–358. 10.11646/zootaxa.5124.3.4 35391119

[B18] Cipola NG Morais JW Bellini BC (2014a) A new species of *Seira* (Collembola: Entomobryidae: Seirini) from Northern Brazil, with the addition of new chaetotaxic characters. Zoologia 31(5): 489–495. 10.1590/S1984-46702014000500009

[B19] Cipola NG Morais JW Bellini BC (2014b) Two new species of *Seira* Lubbock (Collembola, Entomobryidae, Seirini) from South Brazil. Zootaxa 3793(1): 147–164. 10.11646/zootaxa.3793.1.7 24870158

[B20] Cipola NG Morais JW Bellini BC (2016) A new genus of Entomobryinae (Collembola, Entomobryidae) from Brazilian Amazon with body scales and dental spines. Zootaxa 4105(3): 261–273. 10.11646/zootaxa.4105.3.3 27394776

[B21] Cipola NG Morais JW Bellini BC (2017) The discovery of *Lepidocyrtoides* Schött, 1917 (Collembola, Entomobryidae, Entomobryinae) from the New World, including three new species from Brazil and one from Australia. Zootaxa 4324(2): 201–248. 10.11646/zootaxa.4324.2.2

[B22] Cipola NG Arbea J Baquero E Jordana R Morais JW Bellini BC (2018a) The survey *Seira* Lubbock, 1870 (Collembola, Entomobryidae, Seirinae) from Iberian Peninsula and Canary Islands, including three new species. Zootaxa 4458(1): 1–66. 10.11646/zootaxa.4458.1.1 30314142

[B23] Cipola NG Morais JW Bellini BC (2018b) New species, redescriptions and a new combination of *Acanthocyrtus* Handschin, 1925 and *Amazhomidia* Cipola & Bellini, 2016 (Collembola, Entomobryidae, Entomobryinae). Zootaxa 4387(3): 401–435. 10.11646/zootaxa.4387.3.1 29690473

[B24] Cipola NG Morais JW Bellini BC (2020) Review of *Lepidocyrtinus* Börner, 1903 (Collembola, Entomobryidae, Seirinae): The African species. Zootaxa 4898(1): 1–110. 10.11646/zootaxa.4898.1.1 33756843

[B25] Criscuolo A Gribaldo S (2010) BMGE (Block Mapping and Gathering with Entropy): A new software for selection of phylogenetic informative regions from multiple sequence alignments. BMC Evolutionary Biology 10(210): 2–21. 10.1186/1471-2148-10-210 20626897 PMC3017758

[B26] Denis JR (1948) Collemboles d’Indochine récoltes de M. C. N. Dawydoff. Notes d’Entomologie Chinoise 12: 183–311.

[B27] Dierckxsens N Mardulyn P Smits G (2016) NOVOPlasty: De novo assembly of organelle genomes from whole genome data. Nucleic Acids Research 45(4): 1–9. 10.1093/nar/gkw955 28204566 PMC5389512

[B28] Fjellberg A (1999) The Labial Palp in Collembola. Zoologischer Anzeiger 237: 309–330.

[B29] Gisin H (1964) Collemboles d’Europe VII. Revue Suisse de Zoologie 71(4): 649–678. 10.5962/bhl.part.75615

[B30] Godeiro NN Bellini BC (2015) Two new species and two detailed chaetotaxy descriptions of *Seira* (Collembola: Entomobryidae) from Brazil. Zootaxa 3972(2): 208–230. 10.11646/zootaxa.3972.2.4 26249489

[B31] Godeiro NN Zhang F (2021) First record of *Seiradowlingi* (Wray, 1953) (Collembola, Entomobryidae, Seirinae) from China and mitogenome comparison with the New World specimens. Zootaxa 5020(1): 191–196. 10.11646/zootaxa.5020.1.11 34810412

[B32] Godeiro NN Pacheco G Liu SL Cipola NG Berbel-Filho WM Zhang F Gilbert MTP Bellini BC (2020a) Phylogeny of Neotropical Seirinae (Collembola, Entomobryidae) based on mitochondrial genomes. Zoologica Scripta 49(3): 329–339. 10.1111/zsc.12408

[B33] Godeiro NN Zhang F Cipola NG (2020b) First partial mitogenome of a new *Seira* Lubbock species (Collembola, Entomobryidae, Seirinae) from Cambodia reveals a possible separate lineage from the Neotropical Seirinae. Zootaxa 4890(4): 451–472. 10.11646/zootaxa.4890.4.1 33311103

[B34] Godeiro NN Bellini BC Nifeng D Cong X Yinhuan D Zhang F (2021) A mitogenomic phylogeny of the Entomobryoidea (Collembola): A comparative perspective. Zoologica Scripta 50(5): 658–666. 10.1111/zsc.12487

[B35] Haas BJ Papanicolaou A Yassour M Grabherr M Blood PD Bowden J Couger MB Eccles D Li B Lieber M MacManes MD Ott M Orvis J Pochet N Strozzi F Weeks N Westerman R William T Dewey CN Henschel R LeDuc RD Friedman N Regev A (2013) De novo transcript sequence reconstruction from RNA-seq using the Trinity platform for reference generation and analysis. Nature Protocols 8(8): 1494–1512. 10.1038/nprot.2013.084 23845962 PMC3875132

[B36] Handschin E (1925) Beiträge zur Collembolenfauna der Sundainseln. Treubia 6: 225–270. 10.1002/mmnd.48019250305

[B37] Hoang DT Chernomor O von Haeseler A Minh BQ Vinh LE (2018) UFBoot2: Improving the ultrafast bootstrap approximation. Molecular Biology and Evolution 35(2): 518–522. 10.1093/molbev/msx281 29077904 PMC5850222

[B38] Hüther W (1986) New aspects in taxonomy of *Lepidocyrtus* (Collembola). In: Dallai R (Ed.) 2^nd^ International Seminar on Apterygota, 61–65.

[B39] ICZN [International Code of Zoological Nomenclature] (2000) International Commission on Zoological Nomenclature, 4 ed. International Trust for Zoological Nomenclature, London, 306 pp. https://www.iczn.org/the-code/the-international-code-of-zoological-nomenclature/ [Accessed 07 Aug. 2022]

[B40] Katoh K Standley DM (2013) MAFFT multiple sequence alignment software version 7: Improvements in performance and usability. Molecular Biology and Evolution 30(4): 772–780. 10.1093/molbev/mst010 23329690 PMC3603318

[B41] Lartillot N Rodrigue N Stubbs D Richer J (2013) PhyloBayes MPI: Phylogenetic reconstruction with infinite mixtures of profiles in a parallel environment. Systematic Biology 62(4): 611–615. 10.1093/sysbio/syt022 23564032

[B42] Lubbock J (1870) Notes on the Thysanura. Part IV. Transactions of the Linnean Society of London 27(2): 277–297. 10.1111/j.1096-3642.1870.tb00214.x

[B43] Lubbock J (1873) Monograph of the Collembola and Thysanura. Ray Society, London, 276 pp. 10.5962/bhl.title.11583

[B44] Mari-Mutt JA (1979) A revision of the genus *Dicranocentrus* Schött (Insecta: Collembola: Entomobryidae). Agricultural Experiment Station Bulletin 259: 1–79.

[B45] Meng G Li Y Yang C Liu S (2019) MitoZ: A toolkit for animal mitochondrial genome assembly, annotation and visualization. Nucleic Acids Research 47(11): 1–7. 10.1093/nar/gkz173 30864657 PMC6582343

[B46] Minh BQ Schmidt HA Chernomor O Schrempf D Woodhams MD von Haeseler A Lanfear R (2020) IQ-TREE 2: New models and efficient methods for phylogenetic inference in the genomic era. Molecular Biology and Evolution 37(5): 1530–1534. 10.1093/molbev/msaa015 32011700 PMC7182206

[B47] Nguyen TT (2001) Six new species of Collembola (Entomobryidae) from Vietnam. National Centre for Natural Science and Technology of Vietnam 23(1): 21–29.

[B48] Nicolet H (1842) Recherches pour Servir l’Histoire des Podurelles. Neuveaux Mémoires de la Société Helvétiques des Sciences Naturelles 6: 1–88.

[B49] Rambaut A (2010) FigTree version 1.3.1. Institute of Evolutionary Biology, University of Edinburgh, Edinburgh. http://tree.bio.ed.ac.uk/software/figtree/ [Accessed 30 October 2022]

[B50] Schött H (1917) Results of Dr. E. Mjöberg’s Swedish Scientific Expeditions to Australia, 1910-1913. No. 15, Collembola. Arkiv för Zoologi 11(8): 1–60. 10.5962/bhl.part.1499

[B51] Schött H (1925) Collembola from Mount Murud and Mount Dulit in Northern Sarawak. Sarawak Museum Journal 3: 107–127.

[B52] Shorthouse D (2010) Simple Mappr, an online tool to produce publication-quality point maps. https://www.simplemappr.net/ [Accessed 20 September 2022]

[B53] Simon S Letsch H Bank S Buckley TR Donath A Liu S Machida R Meusemann K Misof B Podsiadlowski L Zhou X Wipfler B Bradler S (2019) Old World and New World Phasmatodea: Phylogenomics resolve the evolutionary history of stick and leaf insects. Frontiers in Ecology and Evolution 345(7): 1–14. 10.3389/fevo.2019.00345

[B54] Soto-Adames FN (2008) Postembryonic development of the dorsal chaetotaxy in *Seiradowlingi* (Collembola, Entomobryidae); with an analysis of the diagnostic and phylogenetic significance of primary chaetotaxy in *Seira*. Zootaxa 1683(1): 1–31. 10.11646/zootaxa.1683.1.1

[B55] South A (1961) The taxonomy of the British species of *Entomobrya* (Collembola). Transactions of the Royal Entomological Society of London 113(13): 387–416. 10.1111/j.1365-2311.1961.tb00798.x

[B56] Steenwyk JL Buida III TJ Labella AL Li Y Shen XX Rokas A (2021) PhyKIT: A broadly applicable UNIX shell toolkit for processing and analyzing phylogenomic data. Bioinformatics (Oxford, England) 37(16): 2325–2331. 10.1093/bioinformatics/btab096 33560364 PMC8388027

[B57] Szeptycki A (1979) Chaetotaxy of the Entomobryidae and its phylogenetical significance. Morpho-systematic studies of Collembola. IV. Polska Akademia Nauk, Zakład Zoologii Systematycznej i Doświadczalnej, Państwowe Wydawnictwo Naukowe, Warszawa, Kraków, 219 pp.

[B58] Yoshii R Suhardjono YR (1992) Collembolan fauna of Indonesia and its affinities III: Collembola of Timor Island. Acta Zoologica Asiae Orientalis 2: 75–96.

[B59] Yosii R (1959) Studies on the Collembolan Fauna of Malay and Singapore with special reference to the Genera: *Lobella*, *Lepidocyrtus* and *Callyntrura*. Contributions from the Biological Laboratory Kyoto University 10: 1–65. 10.5134/176436

[B60] Yosii R (1961a) On some Collembola from Thailand. Nature and Life in Southeast Asia 1: 171–198.

[B61] Yosii R (1961b) Phylogenetische Bedeutung der Chaetotaxie bei den Collembolen. Contributions from the Biological Laboratory Kyoto University 12: 1–37.

[B62] Yosii R (1966) On some Collembola of Afghanistan, India and Ceylon, collected by the Kuphe-expedition, 1960. Results of the Kyoto University Scientific Expedition to the Karakoram and Hindukush 8: 333–405.

[B63] Zhang F Deharveng L (2015) Systematic revision of Entomobryidae (Collembola) by integrating molecular and new morphological evidence. Zoologica Scripta 44(3): 298–311. 10.1111/zsc.12100

[B64] Zhang F Chen Z Dong RR Deharveng L Stevens MI Huang YH Zhu CD (2014) Molecular phylogeny reveals independent origins of body scales in Entomobryidae (Hexapoda: Collembola). Molecular Phylogenetics and Evolution 70: 231–239. 10.1016/j.ympev.2013.09.024 24099889

[B65] Zhang F Bellini BC Soto-Adames FN (2019) New insights into the systematics of Entomobryoidea (Collembola: Entomobryomorpha): first instar chaetotaxy, homology and classification. Zoological Systematics 44(4): 249–278. 10.11865/zs.201926

